# Citizen Science, Health, and Environmental Justice

**DOI:** 10.1007/978-3-030-58278-4_12

**Published:** 2020-08-29

**Authors:** Luigi Ceccaroni, Sasha M. Woods, James Sprinks, Sacoby Wilson, Elaine M. Faustman, Aletta Bonn, Bastian Greshake Tzovaras, Laia Subirats, Aya H. Kimura

**Affiliations:** 1 Museum für Naturkunde Berlin – Leibniz, Institute for Evolution and Biodiversity Science (MfN), Berlin, Germany; 2 Faculty of Science, Leiden University, Leiden, The Netherlands; 3 Earthwatch Europe, Oxford, UK; 4 Faculty of Geo-Information Science and Earth Observation (ITC), University of Twente, Enschede, The Netherlands; 5 OpenSystems, Departament de Física de la Matèria Condensada, Universitat de Barcelona, Barcelona, Spain; 6 Department of Applied Information Technology, University of Gothenburg, Gothenburg, Sweden; 7 Department of Bioscience Engineering, University of Antwerp, Antwerp, Belgium; 8 Museum für Naturkunde Berlin – Leibniz, Institute for Evolution and Biodiversity Science (MfN), Berlin, Germany; 9 Earthwatch Europe, Oxford, UK; 10 School of Public Health, University of Maryland, Collage Park, MD USA; 11 Environmental and Occupational Health Sciences, University of Washington, Washington, USA; 12 Helmholtz-Centre for Environmental Research – UFZ & Friedrich Schiller University Jena & German Centre for Integrative Biodiversity Research (iDiv) Halle-Jena-Leipzig, Leipzig, Germany; 13 Center for Research & Interdisciplinarity (CRI), Université de Paris, Paris, France; 14 Eurecat – Centre Tecnològic de Catalunya and Universitat Oberta de Catalunya, Barcelona, Spain; 15 University of Hawai’i, Hawai’i, USA

**Keywords:** Review, Controversies, Social responsibility

## Abstract

This chapter considers the interface of citizen science, health, and environmental justice. We review citizen science research undertaken by civic educators, scientists, and communities that aims to broaden scientific knowledge and encourage democratic engagement and, more specifically, to address complex problems related to public health and the environment. We provide a review of the current state of existing citizen science projects and examine how citizen science, health, and environmental justice impact each other, both positively and negatively. Specific challenges that relate to these projects are discussed, especially those that are not obvious or applicable to more traditional citizen science projects.

## Introduction

This chapter considers work undertaken by civic educators and scientists together with citizen communities to advance health and medical research, to foster scientific literacy, and to encourage democratic engagement. This allows society to deal scientifically with complex modern problems related to human health and environmental justice.

*Citizen science* has been defined in previous chapters and elsewhere as *voluntary engagement in science* (Ceccaroni et al. 2017; Robinson et al. 2018; Haklay et al., this volume, Chap. 10.1007/978-3-030-58278-4_2) and has been primarily undertaken in the environmental domain. As citizen science is a relatively young field, it is necessary to define *health-related citizen science* and *environmental justice* in order to understand the relationships between the three concepts.

Few research domains are as meaningful to the public as *human health*, which should, therefore, be well-positioned for citizen engagement. Health research encompasses a vast range of potential inquiry, much of which is becoming newly accessible, thanks to technology, especially mobile technology. From air-quality testing to DNA sequencing, the opportunities for citizen contribution have grown exponentially (Wiggins and Wilbanks 2019).

### Controversies at the Interface of Citizen Science and Health

Initiatives investigating human health (physical or mental) can be challenging to assess via employing citizen science approaches and employing the ten principles of citizen science (Robinson et al. 2018). Defining projects as citizen science can also be controversial; below we highlight some of the contributory factors – from most controversial to least controversial (Haklay et al. 2020).

- *The level of active engagement*. If engagement or participation is passive, for example, consisting of citizens either as patients or wearing digital sensors, projects tend not to be classified as citizen science.
- *The purpose of knowledge production*. If the goal is mainly commercial (e.g. the development of a drug), projects tend not to be classified as citizen science. Of course, the purpose of knowledge production should never be solely commercial but also related to health improvement.
- *The level of expertise required to participate*. If projects mainly target experts, they tend not to be classified as citizen science.
- *Data sharing*. If data are collected by a commercial enterprise or not shared, projects tend not to be classified as citizen science. While in other domains sharing personal data is sometimes problematic, in the health domain, it is often a prerequisite to participation.
- *The organisational context*. The same activity (such as a trial of a treatment) can be undertaken by a public hospital, a public university, or a commercial actor and assessed by citizens as citizen science if it is conducted by a public organisation, but not if undertaken by a commercial organisation. This assessment is often not justified because attitudes and aims of public and commercial organisations are not necessarily different in practice. As an example, the following is an extract taken from a 2020 public university trial of the ChAdOx1 nCoV-19 vaccine: ‘The results of this research study may be presented at scientific meetings or conferences and published in a scientific medical journal. If you contact the researchers in the future, you can obtain a copy of the results. You will not be identified in any report or publication. The de-identified data from this study will be shared with the collaborating partners who are organising and funding this research work. *Data from this study may be used to file patents, licence vaccines in the future or make profits in other ways* [emphasis added]. You will not be paid for any part of this’ (University of Oxford 2019).
- *Involvement of commercial activities in industry and academia*. Project ethics can be influenced by whether a non-profit or a commercial entity controls the project. While a level of scepticism exists towards business involvement in citizen science, some observe that the sector has made positive and impactful contributions towards advancing the tools and application of citizen science as well as in providing volunteers through campaigns that engage their employees. Finally, organisations from any sector can undertake citizen science projects that do not follow the *ECSA 10 Principles of Citizen Science* (Robinson et al. 2018).

### Environmental Justice

Environmental justice can refer to both the *natural environment* and the *social environment*, with the latter covering aspects of social, economic, and political justice, as well as racism and classism. In this chapter, we analyse both kinds of environment. In relation to the natural environment, environmental justice refers to how ecological degradation (including pollution), landscape destruction, and massive biodiversity loss have the most significant impacts on people on low incomes. An example is when people on low incomes can only afford to live in areas with high levels of ecological degradation or pollution, such as where landfills are located. *Environmental injustice* can take many forms: at a basic level, it includes an unequal burden of environmental hazards (such as landfills, incinerators, polluted sites, industrial livestock production) and unequal access to environmental amenities (such as parks) across geographies, communities, and populations. Environmental injustice most strongly impacts communities of colour (Abara et al. 2012; Wilson 2009).

Environmental justice is not confined to the geographic and demographic distribution of hazards and amenities. It also includes critical political and social processes by which communities can either control their environmental fate or be deprived of control (Holifield 2001).

In this chapter, we consider the fields of citizen science and health (including *public health* and *population health*) with particular reference to the effects on environmental justice. Related work can be driven either by communities or by entities such as universities, public bodies, and commercial organisations. To this end, the chapter begins by considering the relationships and interplay between the fields of citizen science, health, and environmental justice and how they can influence each other both positively and negatively. A review of the current state of play regarding related citizen science projects is then presented, described through a typology that considers tasks, research focuses, and participatory models. The chapter concludes by acknowledging some of the challenges faced by projects that bring together these three disciplines and reflecting on their relevance, trends, and future opportunities.

## The Relationships, History, and Development of Citizen Science, Health, and Environmental Justice

**Fig. 12.1:**
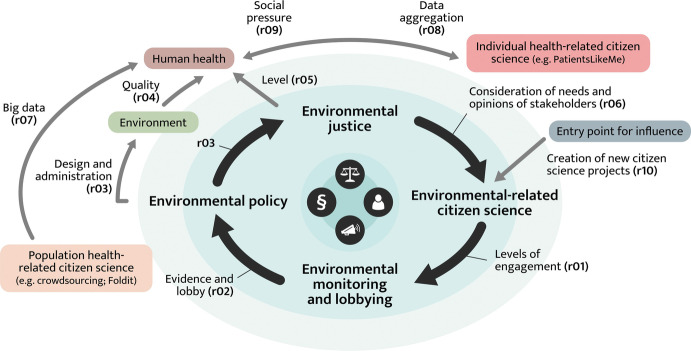
Interactions between citizen science, health, and environmental justice. Relations (rXX) are defined in the text

The links between citizen science, health, and environmental justice are complex. We illustrate these relationships in Fig. 12.1, before providing examples from the literature to substantiate the claims the links represent. We aim to facilitate a better understanding of the role of citizen science, the different ways it enacts this role, and the repercussions for health and environmental justice.

The central ‘ring’ shown in Fig. 12.1, which represents the environmental justice and citizen science concepts and their influences, can be described as a feedback loop, which can be either positive or negative.

In a *positive feedback* scenario, higher levels of engagement in environment-related citizen science activities (**r01**) can lead to increased monitoring of, and lobbying for, environmental issues (Nascimento et al. 2018). The resulting evidence and lobby (**r02**) can lead to new, positive, and measurable environmental policies. Well-designed and well-administered environmental policies can have a positive impact (**r03**) on both the environment itself and environmental justice (Bullard and Johnson 2000). For example, through the fair treatment of all people with respect to the development, implementation, and enforcement of these policies, both a better environment (**r04**) and an increase in environmental justice (**r05**) can have a positive impact on human health (Taylor et al. 2006). Environmental justice, by considering the needs and opinions of a diverse range of stakeholders (**r06**), can further increase engagement and improve the outcomes of environment-related citizen science activities (Shirk et al. 2012). Health can be positively influenced (**r07**) by citizen science initiatives focusing on population health (Candido dos Reis et al. 2015). Health can also exert its influence on (**r08**) as well as be influenced by (**r09**) individual health-related citizen science projects, such as PatientsLikeMe, the world’s largest personalised health network that helps people find new treatments, connect with others, and take action to improve their outcomes (Wicks et al. 2010). By making society ‘fairer’ (**r06**) and by creating new citizen science projects related to environmental issues (**r10**), it is possible to increase environmental citizen science engagement. This can, directly and indirectly, improve the environment, environmental justice, and human health.

A *negative feedback* scenario can also be observed. The absence or reduction of environmental justice can lead to reduced levels of engagement in environment-related citizen science (**r06**). Lower levels of participation in environment-related citizen science (**r01**) can lead to decreased monitoring of, and lobbying for, environmental issues by those directly affected by them (**r02**). This can result in environmental policies that are poorly designed, poorly administered, and inadequate for addressing the needs of the community (**r03**). Inadequate environmental policies can harm both the environment itself and environmental justice (**r03**). Both a compromised environment (**r04**) and an absence of environmental justice (**r05**) can harm human health (Pearce et al. 2010) and, further, can decrease engagement in environment-related citizen science (**r06**). They can also increase engagement, as citizens might be motivated to change the situation. Potentially, then, making society less ‘fair’ not only impairs citizen participation and empowerment but directly and indirectly degrades the environment and human health.

The ‘Entry point for influence’ shown in Fig. 12.1 represents the impact that citizen science can have within these feedback loops. Citizen science can contribute to these relationships in a positive manner. This can be achieved through increased and improved citizen empowerment, data accessibility, public transparency, research relevance, and knowledge production (English et al. 2018).

However, this positive influence is not a given, with issues including lack of engagement, data accuracy, and potential bias constituting barriers that can have a negative impact (Kosmala et al. 2016). Taking a broader definition of environmental justice into account, which includes political and social processes, also reveals potential issues. Questions exist regarding who owns the data (Kish and Topol 2015) and the credentials and motives of who is coordinating the effort (Boulos et al. 2014). These raise issues around misuse of trust and selective inclusion that can be barriers to environmental justice.

### The Relationships Between Citizen Science and Health

Figure 12.1 shows how health can be positively influenced (**r07**) by citizen science initiatives focusing on population health and can affect (**r08**) – and be influenced by (**r09**) – individual health-related citizen science projects, such as PatientsLikeMe and War on Cancer. For example, the War on Cancer project, through an app launched in 2016, offers a safe space where anyone affected by cancer can share stories. Importantly, by collecting this narrative evidence, it can accelerate the search for a cure. The industry has difficulty in obtaining self-reported data on how patients are coping and feeling and how they are responding to treatment. However, most patients are willing to share these data if they understand the purpose. War on Cancer makes money by building tools that allow patients to share their data with researchers and pharmaceutical companies and, crucially, keep the patients informed about the results of that research. There are many patient communities whose activities resemble citizen science or are a form of citizen science. For example, work by the Precision Medicine Initiative (Collins and Varmus 2015) is targeting patient engagement in research and using the ‘citizen science’ label for large-scale research.

### The Relationships Between Citizen Science and Environmental Justice

It is difficult to disconnect environmental justice-related citizen science projects from health. Even if health is not considered to be a central aim of such projects, any engagement in citizen science (be it for the natural or social environment) can be linked to improvements in mental health, through a feeling of purpose and belonging as well as self-actualisation and empowerment (O’Brien et al. 2010). The natural environment itself can have positive effects both mentally and physically when it is the setting for citizen science projects. This connection between environmental justice and health is represented in Fig. 12.1, whereby the central environmental ring and external health concepts are distinct but inextricably linked.

## The Current Landscape

Health-related citizen science projects that address environmental justice are the primary focus of this chapter. Several models exist that reflect the relationship between citizen science, community-engaged research, and research in environmental and public health (Wiggins and Wilbanks 2019). Related activities can be classified depending on the *task type* (data collection or data processing), *research focus* (observational research or interventional research), and *participation models* (including N-of-1 and N-of-we, as defined later in this chapter). We present examples from the literature of how citizen science projects investigating health also relate to environmental justice, using the classification system described by Wiggins and Wilbanks (2019).

### Task Type

Though Wiggins and Wilbanks (2019) admit that having just two categories of task type – *data collection* and *data processing* – somewhat oversimplifies the range of activities that citizen scientists may engage in, this classification is sufficient within the scope of this chapter.

*Data collection citizen science* projects include observational studies of personal health data, the human microbiome,^1^ and pollution. Pollution includes sensory pollution which causes adverse sensory effects in humans by stimulating the senses.

Sensory pollution can be used as a proxy of environmental contamination (Wargocki 2004) and integrated into environmental justice programmes by using environmental sensors. An example is the A Day in the Life programme – a collaboration between the University of Southern California Community Engagement Program on Health and the Environment (USC CEPHE) and three environmental justice organisations – which focuses levels of personal exposure to air pollution and youth engagement. Across California, people of colour are more likely to live near facilities that emit *fine particulate matter* (particles <2.5 μ in diameter, PM_2.5_), a pollutant which increases the risk of cardiovascular disease, respiratory disease, and neurological disorders. Johnston et al. (2019) note that, by providing youth participants with portable personal PM_2.5_ monitors, citizen science can ‘build upon principles of community-driven participatory research, which seeks to deconstruct traditional power dynamics, provide information about environmental hazards important to residents, and democratise knowledge’. This democratisation of knowledge exemplifies how citizen scientists collecting health-related pollution data can address environmental injustice.

Technology can act both as a facilitator and as a barrier to environmental justice in health-based citizen science. In the above instance, the development of a low-cost, low-tech sensor facilitated the creation of an inclusive citizen science project.

Online *data processing citizen science* projects have often relied on gamification approaches to make repetitive tasks more enjoyable, therefore motivating and sustaining participation (Eveleigh et al. 2013). Mechanisms such as league tables, badges, and scoring systems have been used to sustain the engagement of some volunteers; however, others can be alienated by the competitive aspects (Iacovides et al. 2013). As discussed by Newman et al. (2012), while such games and new technologies can appeal to some participants, dependence on them can inadvertently widen the *digital divide* between participants willing and able to adopt the technology and those unwilling or unable to do so.

Data processing and analysis formed the core research activity of the Southern California Environmental Justice Collaborative (SCEJC), an initiative between Communities for a Better Environment, Liberty Hill Foundation, and a multidisciplinary academic research team established to promote environmental health and social justice issues. The SCEJC had two main goals: firstly, to improve environmental health in low-income communities of colour, by conducting citizen science research on air quality, and, secondly, to build the capacity of community-based environmental justice advocacy through training opportunities. The SCEJC applied a citizen science approach to conduct research using *secondary data sources*. This avoided the potential for (misguided) criticism from the scientific community regarding primary data collection quality conducted by citizen scientists. By analysing the data gathered by the government, the SCEJC determined where patterns of environmental injustice existed and which communities suffered potential health impacts as a result. As a result, they were able to demonstrate the effects of cancer-causing air pollutants on communities of colour and to campaign to tighten the standards (Petersen et al. 2006).

### Research Focus

Health-focused citizen science research can be *observational* or *interventional*, while both research types can positively address the issue of environmental justice.

*Observational studies*, in which citizen scientists observe a situation or organism and collect data about it, form the basis for most established citizen science projects. In one observational study, *Tools for Community-based Health Monitoring and Health Impact Assessment – Exploring ‘Citizen Science’ Approaches* (Den Broeder et al. 2017), the perceived impacts of participation in a public health citizen science project on the citizen scientists themselves – in a disadvantaged neighbourhood in the Netherlands – were investigated in order to address environmental injustice. Citizen scientists characterised by low income and educational level were trained to interview fellow residents about health-enhancing and health-damaging neighbourhood features. Observations showed that citizen scientists perceived participation in the project as a positive experience, resulting in acquisition of a broader understanding of health and its determinants and knowledge about healthy lifestyles.

*Interventional studies*, in which an intervention is made during the study, can take the form of citizen science in health and biomedical sciences but are rare in citizen science approaches in other domains. One interventional study (Linking Breast Cancer Advocacy and Environmental Justice) had both political and educational aims. At the *political level*, the study aimed to inform local decisions regarding a nearby oil refinery, state policies regarding chemicals, and political decisions regarding *endocrine-disrupting compounds* (EDCs)^2^ in consumer products. At the *educational level*, the project aimed to inform community members about the determinants of their indoor and outdoor air quality, strategies to reduce their exposure to pollutants, and the potential implications of contaminants on community health. The study resulted in increased environmental health education, which subsequently stimulated further public involvement and changes in community behaviour. Moreover, and most noteworthy, the project resulted in a legal victory that blocked the expansion of the oil refinery. This decision not to expand the refinery was considered a *public health intervention*, supporting our ontology: lobbying for the environment via citizen science initiatives leads to increased environmental justice and improved public health (Fig. 12.1, **r02**, **r03**, **r04**).

A second example of interventional health-related citizen science addressing environmental justice is the Our Voice initiative, led by Stanford Medicine, which empowers communities to make a positive impact on their local environment. Our Voice works with research institutions and community-based organisations around the world to (1) encourage citizen scientists to discover which aspects of their surrounding environment have an impact on healthy living; (2) support them to discuss their findings with other citizen scientists; and (3) enable them to change their community (including natural and social environments and health) for the better. In one such partnership with GirlTrek – a civil rights-inspired health movement encouraging African American women to adopt a daily habit of walking as a way to reclaim their neighbourhoods – citizen scientists across eight cities were trained in the Our Voice Discovery Tool mobile app. This resulted in 230 photographs being analysed to assess neighbourhood features that improve walkability. As a direct consequence of the project, sidewalks were repaved around an elementary school, and the length of time for pedestrians to cross the road at a crosswalk was increased from 20 to 40 seconds.

### Participation Models

The participation models considered in this chapter are *N-of-1* and *N-of-we*. While there are other models discussed by Wiggins and Wilbanks (2019), these two lend themselves most naturally to health-based citizen science initiatives related to environmental justice.

#### N-of-1

In medicine, an N-of-1 trial is a clinical trial in which a single patient is the entire trial or case study. Examples are data collection of one’s daily actions, the possible analysis of those actions, and the observation of outcomes in response to interventions. N-of-1 can include *self-tracking*: individual-driven, personal experiments sparked in part by the growing ease of collecting data, reporting data, and analysing data. An example is using wearables to track heart rates. *Generalised N-of-1* is a project in which a single citizen collects or analyses scientific observations of any kind, not necessarily about themself. These studies are more individualistic than other citizen science projects. Since citizen science is primarily associated with collective models of participation, generalised N-of-1 studies are less likely to be recognised as citizen science unless they become visible through coordination or sharing of results.

In this sense, one of the most famous examples of generalised N-of-1 environmental justice studies related to health involved the collection of landfill data. The study found that, between the 1930s and the 1970s, 80% of all the waste in the Houston area was dumped in neighbourhoods predominantly made up of communities of colour. This practice was neither random nor isolated to Houston, with targeted and widespread injustice demonstrable across the southern states of the USA. There is evidence to suggest that living within 5 km of a landfill is associated with increased mortality from lung cancer and respiratory disease. Thus, environment-based citizen science, to monitor the natural environment and improve environmental policy to ease environmental injustice, also feeds into human health.

Environmental injustice can be subtler than the placement of landfills and oil refineries; it can manifest itself as negligence. The lack of action can lead to the development of less ‘walkable’ locations. At least in the USA, such locations are related to less-active residents, who are more likely to be obese, with increased risks of high blood pressure, high cholesterol, heart disease, and stroke. While public health studies have linked socioeconomics and race to the risk of obesity, these studies do not take factors such as marginalisation and disinvestment (issues of environmental justice) into account.

#### N-of-we

In N-of-we models, N-of-1 data sets are connected to form a more general knowledge base. The work related to these models is often community driven or public driven. One example is the citizen science project Mosquito Stoppers, funded by the National Science Foundation in the USA, that studies the exposure to mosquito-borne pathogens. Effective control of mosquito populations and of the diseases they carry requires explicit spatial knowledge about their habitat; citizen science projects can provide this knowledge. Project leaders established four priorities: (1) making open spaces healthy and appealing; (2) alleviating the burden of mosquito exposure in disinvested communities; (3) reinvestment in disinvested communities with substantial participation by residents; and (4) improvement of city sanitation services. The first priority could arguably be seen to also address mental health, as spending time outside has been demonstrated to improve health and well-being.

In communities facing environmental injustice, unmanaged infrastructures, a lack of redevelopment, and the often-associated build-up of waste (due to limited waste collection services through disinvestment) contribute to higher adult mosquito density because they provide a more favourable habitat. These communities have lower health levels and are less likely to be engaged in citizen science.

To promote the co-management of the project, citizen science leaders were recruited from within the community; citizen knowledge was incorporated via two channels (mosquito population data collection and qualitative citizen science experience data); and the results were disseminated at neighbourhood meetings. Community members were encouraged to contact city services using data on waste issues throughout their neighbourhoods (‘calling to report trash and request the city to clean it up’), as part of translating data to on-the-ground outcomes (Sorensen et al. 2018).

On paper, this health-related citizen science project directly addressed environmental justice to drive action and change. Nevertheless, even such a well-conducted and well-meaning project is not without its challenges. It was noted that many participants began to express fatigue, as they felt increasingly frustrated that they kept noticing, and reporting, the same piles of waste and the same abandoned buildings, but nothing was ever done by the authorities. The problem of those in power not acting on the data generated by those lacking power is one of several challenges we encountered while working on this chapter. These challenges are the focus on the next section.

## Challenges

### Addressing Health Disparity

*Health disparity*, the gap between the health of the rich and the health of the poor, is a significant issue. If the rich use their superior health to enrich themselves further, and if more money can buy them enhanced bodies and brains, with time, this gap will only widen. Soon, the wealthiest 1% might own not only most of the world’s wealth but also most of the world’s health. Factors to consider are the reduction in government spending on public health, the shrinking investment in the treatment of diseases that hit the marginalised in society, as well as the cost of diagnostic tests and procedures. As discussed in a prior section, health-related citizen science projects do not always directly address environmental justice. Are, then, current health-related citizen science projects that do *not* directly address environmental justice unwittingly raising barriers to inclusion, and, if so, what can be done to remove these barriers? To make an already problematic situation even worse, as the masses lose their economic and political power, the state has less incentive to invest in their health. This can be observed in the laissez-faire response from the UK government in March 2020 to COVID-19 (ICD-10 B97.2 and U07.1), a disease killing mainly those with low levels of economic and political power (Bialek et al. 2020).

When scientists are confronted with this scenario, their standard reply is that many medical breakthroughs begin with the rich but eventually benefit the whole population and help to narrow rather than widen social gaps. For example, vaccines and antibiotics initially profited mainly the upper classes in the Global North, but today they improve the lives of humans globally. However, the expectation that this process will be repeated in the twenty-first century may be just wishful thinking, for two crucial reasons. First, medicine is undergoing a *conceptual revolution*. Medicine in the Global South (e.g. in China or in most of Africa) aims to heal the sick, while medicine in the Global North increasingly (e.g. in the UK) seeks to enhance the healthy. For example, a report suggests that life expectancy in the UK has stalled for the first time in more than a 100 years and is in decline for the most deprived women in society (Marmot 2020). Second, medicine in the Global South benefits the masses because the Global South is home to the masses. Armies in the Global South need healthy soldiers, and the economy requires healthy workers. These needs do not exist in the Global North, or else will soon no longer exist.

Nevertheless, citizen science programmes on, for example, air pollution can lead to policy measures to improve air quality, from which everybody will benefit, not only the rich.

### Gaps in the Ability to Volunteer

We also need to consider that citizen science is volunteer based and how that relates to *time poverty* (the idea that discretionary time is class based). Kimura and Kinchy (2019) discuss the dilemmas related to the class stratification of *volunteerism* (which has become popular under neoliberalism) in more detail.

### Neoliberal Transfer of Responsibility

Furthermore, we should consider if citizen science practices reinforce the *neoliberal transfer of responsibility.* Citizen science needs to be situated in the broader dynamics of neoliberalism, where accountability for health and well-being is increasingly individualised. Indeed, it is not a coincidence that the growth of citizen science coincides with lower governmental spending on environmental monitoring, health, and scientific research. Citizen science is effective in providing fine-grained data that considers local/personal knowledge. Yet, such personal-level attention can shift the scale at which health or environmental problems are conceptualised, from social/structural to individual. Citizen science needs to navigate the challenging situation in which collecting data can sometimes reinforce the neoliberal transfer of responsibility to citizens (Kimura 2016; Kimura and Kinchy 2019).

### Privacy

Health-related citizen science projects often face challenges around *privacy*. Health information is very sensitive, so health-related citizen science initiatives should bear this in mind and explore appropriate modes of data governance.

### Interoperability

Communication (multilingual and interdisciplinary) is another challenge that health-related citizen science projects face. The use of international standards and vocabularies is essential to cover a global perspective and allow data from different countries to be aggregated, studied, and compared. In health, some of these standards and concepts are promoted by the World Health Organization (WHO):

- International Classification of Diseases (ICD)^3^
- International Classification of Functioning, Disability, and Health (ICF)
- International Classification of Health Interventions (ICHI)
- Anatomical, Therapeutic, Chemical Classification System (ATC)^4^
- Disability-adjusted life year (DALY)^5^

Even when collecting interdisciplinary data according to international standards, health-related citizen science projects can face challenges in addressing environmental injustice. Quantifying the health risks of exposure to a single toxic compound is inherently problematic in terms of being able to isolate its effects from other environmental factors. Thus, while there are established correlations between environmental exposure to particular chemicals and particular diseases, the levels of exposure (in terms of concentration and duration) and how best to measure these are continually disputed. This makes deriving solutions from such data even more problematic.

While providing answers to such issues is beyond the scope of this chapter, we recognise the importance of organisations putting pressure on governments and other institutions to include under-represented groups and interests in health-related citizen science projects. We recommend that all citizen science projects seek opportunities to reach citizen scientists from different classes. We will discuss inclusivity further in the final section of this chapter.

Defining the interplay between the three (not immediately obviously related) concepts of citizen science, health, and environmental justice is challenging. Despite the use of citizen science since the 1990s, its utility in health research is relatively novel, health-based citizen science as a way of addressing environmental justice even more so. Interdisciplinary environments are themselves challenging. Experts from several disciplines need to work together to reach a common goal; apart from using the same vocabulary, they need to share knowledge, processes, and best practices.

The definition of environmental justice itself has proven controversial. While we have predominantly focused on the natural environment in this chapter, we acknowledge that environmental justice is also a social issue that extends to the economic and political contexts. Citizen science, if used *to empower additional people to join the debate about the future*, undoubtedly has a role to play in driving the changes necessary to facilitate social and political environmental justice. However, citizen science is naturally suited to research on the natural environment, more so than to issues such as the economy.

### Disparity and Power Imbalance

It is important to consider the specific challenges for citizen science in the Global South, where citizen science might be driven by foreign international organisations as a part of their data gathering or development work.

First, *who asks the questions?* Whether the scientific questions to be addressed by a citizen science project come from citizens themselves or civic educators (including scientists, professional researchers, and their institutions) needs to be interrogated. Is it reasonable to expect communities to initiate and drive a socio-environmental-justice movement? Is it overambitious to assume that they *can*? Does suggesting that they *cannot* indicate a level of institutional and structural racism? Empowering people includes enabling them to initiate citizen science projects. Nevertheless, in most cases an external organisation is the initiator. In the field of health-related science, the history of modern Western medicine connects seamlessly with that of European colonial expansion in the nineteenth century. Quinine enabled European armies to enter previously forbidden terrains. Medical officers helped to sanitise dangerous spaces and environments but also subjected Indigenous populations to European rule. Today, as in the past, efforts to curb epidemic and pandemic diseases such as plague, smallpox, cholera, and COVID-19 lead to attempts to discipline the routines, diets, and movements of citizens. Effective medical interventions and vaccination programmes help to maintain a healthy labour force (Keller 2006). In this context, scientific questions are asked by people in power: only their knowledge counts, and the science, while presented as a benefit to the citizens, is used as an effective means for control. Ultimately, science is for the people in power, not the citizens. Therefore, whenever science is being conducted in the Global South, it is pertinent to additionally consider:

- Whose knowledge counts when asking the question?
- How is science being used?
- Who, ultimately, is the science for?

Second, *does citizen science marginalise Indigenous knowledge?* Whether citizen science inadvertently cements such historical dynamics of marginalisation is a question that needs to be investigated. A considerable part of environmental justice science deals with the *colonisation of science*: the disproportionate legacy of white European thought and culture in science. Modern Western science is inextricably linked with colonialism, especially British imperialism. The scientific successes of the West were used to allege that non-Westerners were intellectually inferior and so deserved and needed to be colonised. Although colonialism has formally ended, these attitudes have not yet wholly disappeared. Academic journals are dominated by Western papers, stemming from the top-ranking universities, because the scoring system is Western. A study of papers produced by central African countries revealed that 80% of the region’s output was produced in collaboration with a partner from outside the area, with 35% in partnership with past colonial rulers (Boshoff 2009). Attitudes expressed by academics from the Global North towards academics from the Global South are sometimes alarming. They suggest that attitudes expressed by academics from the Global North towards mere citizens from the Global South could be even worse (Dahdouh-Guebas et al. 2003).

Third, *does citizen science have a significant impact on justice?* Environmental justice is not focused on documentation and observation but on action, and citizen scientists investigating their environments (natural or social) do not need academics or researchers to validate the science they undertake. Though scholars and other professional scientists are undoubtedly interested in the issues of environmental justice, and are keen observers, they are generally not as motivated to drive improvements in unjust environments as the communities themselves. Therefore, there is a lack of focus on generating science that translates results into action, indicative of the issues of power and control.

Fourth, *does citizen science drive democratisation?* A key challenge is how to democratise science, whether it be science for health or science for environmental justice. Citizen science needs to acknowledge the diversity of participants in terms of language and literacy and address the issue of *who conducts science?* For example, the Northern California Household Exposure Study, by encouraging women and people of colour to present the results of their study at community meetings in both English *and* Spanish, was able to at least challenge (if not change) ideas about who conducts science.

## Relevance, Future Trends, and Recommendations

### Relevance and Future Trends

Citizen science, health, and environmental justice are closely linked. For example, pollution has a disproportionate effect on the health of minorities: unequal environmental quality exacerbates social inequality.

Research led by patient communities is an excellent example of user-driven studies and the power of citizen science. If the success of these initiatives can also be adopted in other fields for truly co-created citizen science projects, this will facilitate innovation in science and at the science–society interface.

Understanding how engagement in citizen science itself can provide health benefits – either through (1) experience of self-efficacy and sense of purpose; (2) actual or virtual social contact and cohesion; or (3) being in natural environments – is to date unexplored and presents an essential further avenue of research.

The global environmental changes facing us today are increasingly being recognised as critical, so are issues of environmental justice, as future trends in population growth are linked to food and health equality and to the overuse of the environment. In the Global North, there is a trend towards more individual health-related citizen science. For example, Project Baseline aims to make it easy and engaging for citizens to contribute to the map of human health and participate in clinical research. Together with researchers, clinicians, engineers, designers, advocates, and volunteers, the project contributes to building the next generation of health-care tools and services. Citizens can contribute through clinical research, surveys, and focus groups. They are the first to know when *studies matching their preferences* are launched. They can test new tools, technologies, and treatments and shape the future of health care. In addition, citizens can learn about their own health and simultaneously help improve health for all.

### Recommendations

Barriers to inclusion are a concern in citizen science in general (Paleco et al., this volume, Chap. 10.1007/978-3-030-58278-4_14) and particularly in health-related citizen science. Therefore, we recommend incorporating *inclusivity* into health-based citizen science project design. Some projects give citizens a great deal of control, while others give credentialed scientists the lead, relegating citizens to a prescribed role. The degree of community involvement varies and changes over time, and project partners need to consider whether the limits placed upon citizen involvement are justified. Moreover, ‘citizens’, ‘communities’, and ‘local people’ are not homogenous. Participants in health-related citizen science are overwhelmingly well-educated, wealthy, and of European ancestry (Greshake Tzovaras and Tzovara 2019). Whether participants genuinely reflect the diverse opinions and lived experiences of those experiencing environmental and health struggles needs to be considered. Citizen science is in a strategic position to ensure the following challenges are addressed. How can we make sure that those with less power, women, and minorities are among those who are asking the questions? Is the science being conducted by a diverse group of people? Is it being analysed by a diverse group of people, using technology that does not discriminate?

Additionally, there are pervasive *biases* in health data; the different types of biases are outlined in the Catalogue of Bias established by the University of Oxford (https://catalogofbias.org/). Citizens and scientists who are analysing collected data must be made aware of these explicit and implicit biases. We recommend that citizen science projects take specific measures to ensure that the data they work with are unbiased and that the algorithms they use are *fair by design*.

In terms of the *role of the business sector*, we note that there is an increasing awareness about the use of citizen science for public relations. Given citizen science’s appeal as a community-based participatory endeavour, there is the possibility that commercial actors will deploy it in a way that enhances their public image, softens community health concerns, and obfuscates, rather than clarifies, their environmental or health impacts. Partnering with the commercial sector also raises issues about the ownership of data and equitable profit sharing. We recommend undertaking research in order to understand the role of the commercial sector and the way it engages with citizen science.

Also, we recommend a citizen science approach that welcomes questions asked by those who are under-represented or lacking in power. Appropriate questions asked by the commercial sector can have positive effects on health-related citizen science projects.

We recommend using citizen science to *empower* additional people to join the debate about the future. Citizen science projects should allow communities that might otherwise be overlooked to drive both the design and the implementation. To ensure the project is genuinely community led, open discussions should be held on how the tools used should function, how they will be used and managed by the community, how the data will be stored, and what the expected outcomes will be. In the case of environment-related citizen science projects, benefits to mental health are apparent, even before data are collected, if empowerment within the community is evident. Community-initiated projects demonstrate that professional scientists are not a prerequisite for science to be conducted and that there is value in empowering citizens to lead scientific endeavours.

Interestingly, citizen science itself could also be seen and evaluated as a health intervention, if it is empowering and thereby engendering a sense of community, social cohesion, as well as self-efficacy. These outcomes could have a salutogenic effect on mental health and well-being for the participants. This effect may, of course, not only apply for health-related citizen science projects but also for citizen science projects in general.

We recommend taking into account *Indigenous and local knowledge* in citizen science projects. While scientific knowledge is validated through peer review by other scientists, other knowledge systems can have different validation approaches which should be considered.

Finally, we recommend considering how international health-related *standards and vocabularies* can be incorporated in a user-friendly way.

We hope this chapter provides a basis for discussion for all those interested in health-related citizen science which aims to address environmental justice and pointers on how to strive for equality and promote positive, sustained impact.

## Footnotes

1 The human microbiome is the aggregate of all microbiota that reside on or within human tissues and biofluids, along with the corresponding anatomical sites in which they reside.

2 EDCs are compounds which affect the endocrine system, which is responsible for metabolism, growth, and development in humans.

3 The latest versions of the ICD, ICF, and ICHI are available from the WHO: https://www.who.int/classifications/en/

4 https://www.whocc.no/atc_ddd_index/

5 https://www.who.int/healthinfo/global_burden_disease/metrics_daly/en/
